# Simultaneous Pancreas and Kidney Transplantation in a Patient With Situs Inversus Abdominalis

**DOI:** 10.7759/cureus.73271

**Published:** 2024-11-08

**Authors:** Christoph Eckharter, Jose Oberholzer, Ricarda Stolzmann-Hinzpeter, Fabian Rössler

**Affiliations:** 1 Department of Surgery and Transplantation, University Hospital Zürich, Zürich, CHE; 2 Department of Radiology, University Hospital Zürich, Zürich, CHE

**Keywords:** first report, pancreas transplantation, simultaneous pancreas and kidney transplantation, situs inversus abdominalis, technical challenges

## Abstract

Simultaneous pancreas and kidney transplantation (SPK) represents the most effective treatment for selected patients with type 1 diabetes and end-stage kidney disease. However, SPK is a technically complex surgical procedure and is particularly challenging in patients with anatomical variations. Situs inversus abdominalis (SIA) is a rare hereditary condition in which the abdominal organs and vessels are arranged in a mirror image of standard anatomy. In this case report, we describe a successful SPK in a patient with SIA. We discuss the surgical technique in detail, especially the importance of correct pancreas graft placement to avoid dangerous complications such as vascular thrombosis.

## Introduction

Simultaneous pancreas and kidney transplantation (SPK) is the gold standard of treatment for selected patients with type 1 diabetes (T1DM) and end-stage kidney disease. Patients who undergo SPK demonstrate a markedly improved long-term survival rate in comparison to those who continue on dialysis or undergo kidney transplantation (KT) alone [1,2]. Patient survival after SPK even exceeds the results after living donor KT after four years [3]. However, SPK is a technically complex and challenging surgical procedure, which is performed on patients with severe comorbidities and bears a considerable risk for technical complications [4].

Situs inversus (SI) is a rare hereditary condition, with an incidence of 1 to 10,000 live births. The term "situs inversus viscerum" is derived from Latin, meaning "inverted position of the internal organs". In this condition, the arrangement of organs and vessels is mirror-inverted to the standard anatomy. However, this does usually not result in any significant functional restrictions. The severity of inversion can be classified as total, involving both the thoracic and abdominal regions, or partial, in which only organs situated above or below the diaphragm are affected [5].

Reports have previously demonstrated the feasibility of liver and kidney transplantation in patients with SI [6,7]. However, to date, there is no report on SPK in patients with situs inversus abdominalis (SIA).

Herein we present the first case of SPK in a patient with SIA, with details on surgical technique, intra- and postoperative course, and graft function.

## Case presentation

**Figure 1:**
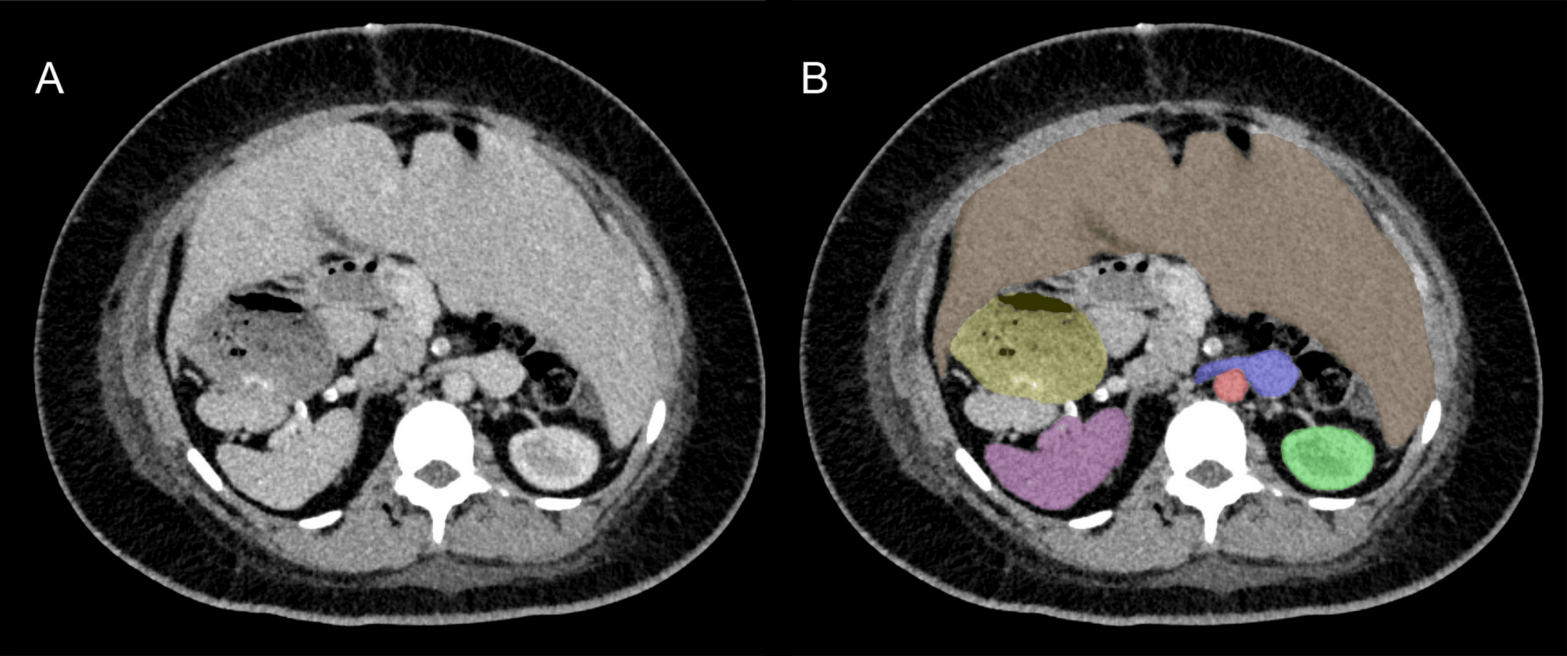
Computed tomography (CT) scan illustrating the mirror-inverted arrangement of abdominal organs in our patient with situs inversus abdominalis. Part A presents the native CT scan, while Part B uses color-coding to enhance organ visibility: liver (brown), stomach (yellow), spleen (violet), aorta (red), vena cava with right kidney vein (blue), and left kidney (green).

Our patient was a 29-year-old woman with known SIA, diagnosed with T1DM at the age of 10. Hemoglobin A1c (HbA1c) values were above 14% and she developed rapid deterioration in kidney function, necessitating the initiation of hemodialysis by the age of 25 years. At the time of transplantation, HbA1c values were around 7.4% (reference range: 4.4 - 5.6 %) with insulin pump therapy. Apart from a body mass index (BMI) of 32.8 kg/m^2^, she was in good clinical condition and her time on the waitlist was 1.7 years. Computed tomography (CT), which was routinely performed during the waiting period, demonstrated the expected anatomy of SIA (Figure 1). This included the spleen and stomach on the right side, the liver on the left side, and the inferior vena cava to the left of the aorta.

**Figure 2:**
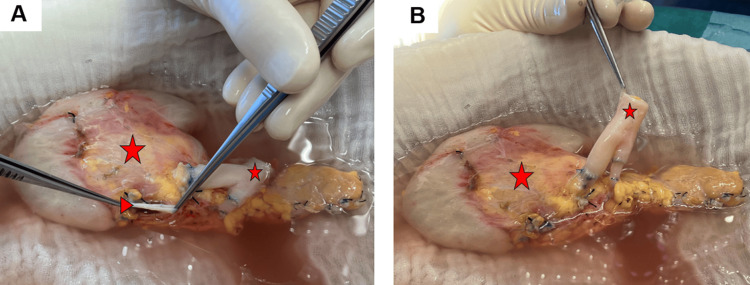
Parts A and B show the pancreas graft on the back table. The arterial Y-graft from the donor iliac artery bifurcation has been connected to the splenic artery and superior mesenteric artery of the pancreas graft. Small asterisk: Arterial Y-graft connected to superior mesenteric artery and splenic artery of the pancreas graft; large asterisk: pancreas graft; arrowhead: portal vein of the pancreas graft

Both grafts were from a 35-year-old male donor after brain death (DBD) due to traumatic brain injury. Procurement of the pancreas was according to our standard, en-bloc with the duodenum and spleen. Transportation was by static cold storage. Back table preparation of the pancreas was according to the previously described standard of our department [8]. We used an arterial Y-graft from the donor iliac artery bifurcation to reconstruct the graft`s superior mesenteric artery (SMA) and splenic artery (SA). The donor external iliac artery was anastomosed to the SMA and the donor internal iliac artery to the SA (Figure 2). After midline laparotomy, detailed inspection of the abdomen revealed the expected anatomy as seen on preoperative CT. Of note, the sigmoid colon was on the left side and the cecum in the middle of the abdomen, corresponding to an incomplete intestinal rotation.

**Figure 3:**
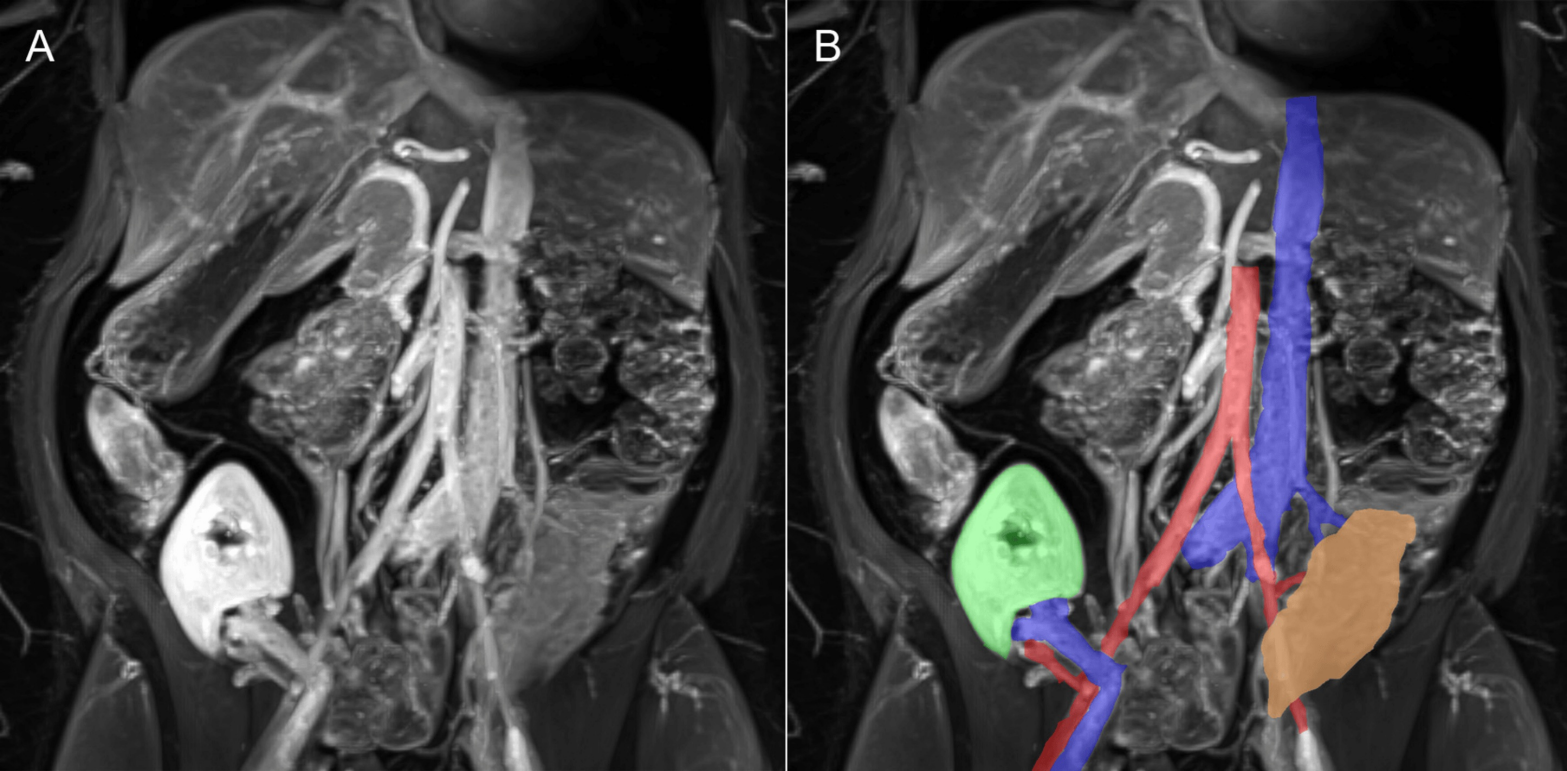
Magnetic resonance angiography (coronary view) following SPK. The pancreas graft is situated in the left iliac fossa in a "head-up" orientation, with its arterial Y-graft connected to the left common iliac artery and its portal vein anastomosed to the inferior vena cava. The renal graft is positioned in the right iliac fossa and connected to the right external iliac vessels. Part A shows the unmodified MRA scan, while Part B features color-coded enhancements to improve organ visibility: pancreas graft (golden yellow), kidney graft (green), aorta and iliac arteries with graft vessels (red), and vena cava and iliac vessels with graft vessels (blue). SPK: Simultaneous pancreas and kidney transplantation

Our current standard technique for SPK is a “head-down” placement of the pancreas graft in the right hemiabdomen and the kidney graft on the ipsilateral iliac axis. The venous drainage of the pancreas is systemic, via the inferior vena cava. The arterial anastomosis is performed via a Y-graft to the right common iliac artery. Enteric drainage is via a side-to-side duodeno-ileostomy. As the inferior vena cava was located to the left side of the aorta, we decided to implant the pancreas graft “head-up” and to the left side of the hemiabdomen. An end-to-side porto-caval anastomosis was performed using Prolene 6-0 with continuous suture. Following venous anastomosis, the arterial Y-graft was anastomosed to the left common iliac artery in an end-to-side position using Prolene 6-0 with continuous suture. After reperfusion and careful hemostasis, the graft`s duodenum was anastomosed to the terminal ileum with a double-row side-to-side anastomosis using a PDS 4-0 suture on the inside and a Prolene 4-0 suture on the outside. Due to the limited space on the left iliac axis resulting from the pancreatic tail, the kidney was implanted on the contralateral side. The renal vessels were connected to the external iliac vessels (Figure 3). Uretero-cystostomy was performed analogous to Lich-Gregoir, and the donor ureter was splinted with a pigtail catheter [9].

The intra- and postoperative courses were uncomplicated, and the patient was discharged from the hospital after nine days. The surgical time was 360 minutes, with a cold-ischemia time of 630 minutes for the pancreas and 760 minutes for the kidney. Anastomotic times were 28 and 44 minutes for the pancreas and kidney, respectively. Pancreas and kidney graft functions were excellent right from the beginning. The patient had been permanently insulin-free since pancreas reperfusion and showed stable plasma glucose levels between 5 and 8 mmol/l on a normal diet. Kidney graft function was immediate and reached a glomerular filtration rate (eGFR) of 34 ml/min (normal range: 90 or higher) according to the Chronic Kidney Disease Epidemiology Collaboration (CKD-EPI) [10] equation at the time of discharge. At three months follow-up, our patient was without complaints, with a HbA1c of 5.6% and an eGFR of 70 ml/min.

## Discussion

This is, to the best of our knowledge, the first report of SPK in a patient with SIA. We address the special considerations and technical challenges associated with SIA in this complex abdominal transplantation. The combined transplantation of the pancreas and kidney was technically successful, without major complications and persistent graft function.

Due to the atypical anatomical conditions, SIA was even considered a contraindication for liver transplantation (LT) in the past. However, since the first successful LT in a patient with SIA in 1988, several more followed [6,11]. In KT, where grafts are usually placed in the iliac fossa, surgeons are used to implant kidney grafts on both sides, using either a left or right donor kidney. The crossing of the recipient's iliac vessels prior to anastomosis permits the parallel positioning of the donor vessels, which may prevent subsequent twisting of the graft vessels or avoid pressure from the artery on the renal vein. It is also our standard approach to shift the external iliac artery medially before implanting a left kidney on the right iliac axis and vice versa. For this reason, KT in patients with SIA is no different than in patients with standard anatomy [5,7]. It is noteworthy that in our patient with SIA, the external iliac vessels were again positioned in a standard manner, with the vein located medially.

In contrast to KT, placement of the pancreas graft can be challenging in patients with SIA. Complicating, the portal vein of the graft is usually kept very short to avoid kinking and subsequent thrombosis, thus not much movement of the graft is possible. Several techniques have been described for graft placement in SPK, with the most important ones being “head-up” and “head-down” for systemic and enteric drainage [12]. In the past, we placed pancreas grafts always “head-up” and to the right side, we adapted this to “head-down”. This allows for better vessel positioning and the possibility to place the kidney on the ipsilateral iliac axis, in order to spare the contralateral axis for subsequent KT.

**Figure 4:**
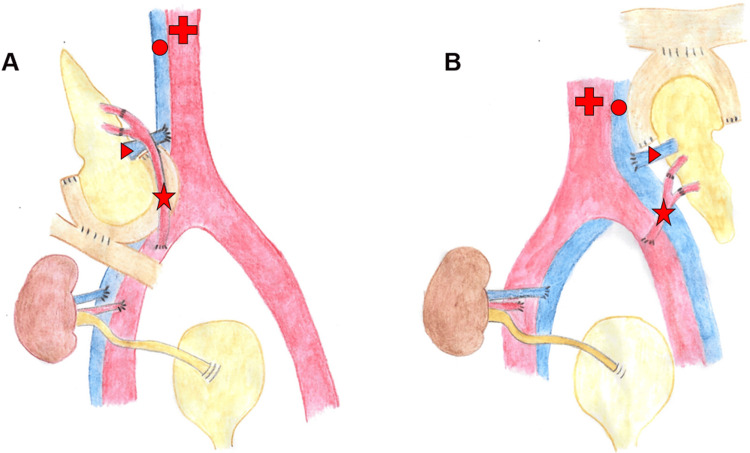
Schematic representation of a simultaneous pancreas and kidney transplantation. Part A for standard anatomy; pancreas graft placed "head-down" in the right hemiabdomen with ipsilateral kidney graft. Part B for situs inversus abdominalis; pancreas placed "head-up" in the left hemiabdomen with contralateral kidney graft. Asterisk: Arterial Y-graft connected to superior mesenteric artery and splenic artery of the pancreas graft; arrowhead: portal vein of the pancreas graft; cross: abdominal aorta; circle: inferior vena cava Image Credits: Christoph Eckharter

The reversed position of the abdominal vessels in SIA, with the aorta on the right side and the inferior vena cava on the left side, required an adaptation of our standard technique. Instead of placing the pancreas graft "head-down" on the right side, we put it "head-up" on the left side of the pelvis to ensure the vessels were parallel. The portal vein was connected to the recipient’s inferior vena cava and the arterial Y-graft to the recipients' left common iliac artery (Figure 4). This position avoids crossing the arterial Y-graft with the donor portal vein, which may be a risk factor for pressure-related venous thrombosis.

Enteric drainage was according to our standard, via the graft`s duodenum to the terminal ileum. Given the location of the cecum in the middle of the abdomen, it was readily accessible, allowing for the placement of the anastomosis 100 cm orally from the ileo-cecal junction. The left donor kidney was ultimately implanted in the right iliac fossa, primarily due to a lack of space in the left iliac fossa caused by the tail of the pancreas graft positioned "head-up."

## Conclusions

This case report describes the first successful SPK in a patient with SIA, with particular emphasis on the technical challenges associated with the vascular anatomy and positioning of the pancreas graft. We recommend a preoperative CT scan in all patients with SIA to evaluate the vascular anatomy in detail. With careful preoperative evaluation, SPK is safe and feasible in patients with such rare anatomical variations.
